# Copresence With Virtual Humans in Mixed Reality: The Impact of Contextual Responsiveness on Social Perceptions

**DOI:** 10.3389/frobt.2021.634520

**Published:** 2021-04-12

**Authors:** Daniel Pimentel, Charlotte Vinkers

**Affiliations:** ^1^ Oregon Reality Lab, School of Journalism and Communication, University of Oregon, Portland, OR, United States; ^2^ Magic Leap, Plantation, FL, United States

**Keywords:** mixed reality, virtual human, spatial computing, agents, copresence, social presence

## Abstract

Virtual humans (VHs)—automated, three-dimensional agents—can serve as realistic embodiments for social interactions with human users. Extant literature suggests that a user’s cognitive and affective responses toward a VH depend on the extent to which the interaction elicits a sense of copresence, or the subjective “sense of being together.” Furthermore, prior research has linked copresence to important social outcomes (e.g., likeability and trust), emphasizing the need to understand which factors contribute to this psychological state. Although there is some understanding of the determinants of copresence in virtual reality (VR) (cf. Oh et al., 2018), it is less known what determines copresence in mixed reality (MR), a modality wherein VHs have unique access to social cues in a “real-world” setting. In the current study, we examined the extent to which a VH’s responsiveness to events occurring in the user’s physical environment increased a sense of copresence and heightened affective connections to the VH. Participants (*N* = 65) engaged in two collaborative tasks with a (nonspeaking) VH using an MR headset. In the first task, no event in the participant’s physical environment would occur, which served as the control condition. In the second task, an event in the participants’ physical environment occurred, to which the VH either responded or ignored depending on the experimental condition. Copresence and interpersonal evaluations of the VHs were measured after each collaborative task *via* self-reported measures. Results show that when the VH responded to the physical event, participants experienced a significant stronger sense of copresence than when the VH did not respond. However, responsiveness did not elicit more positive evaluations toward the VH (likeability and emotional connectedness). This study is an integral first step in establishing how and when affective and cognitive components of evaluations during social interactions diverge. Importantly, the findings suggest that feeling copresence with VH in MR is partially determined by the VHs’ response to events in the actual physical environment shared by both interactants.

## Introduction

Recent advancements in artificial intelligence (AI) and mixed reality (MR) hardware have enabled what industry experts are dubbing as “the age of the virtual human” (Titcombe et al., 2020). Virtual humans (VH) are automated, computer-generated embodied agents capable of a wide range of human behavior (Lucas et al., 2017). Despite their artificial nature, VHs are largely perceived as social actors in part because of their ability to respond realistically to external cues, including users’ affective states (Nass and Moon, 2000; Becker-Asano and Wachsmuth, 2010). This capability has contributed to their integration into social environments, serving as educators in classrooms (Li et al., 2016), companions in homes (Krämer et al., 2015), and medical staff in hospitals (Gunn et al., 2020), among others. Yet, studies on the efficacy of VHs in such contexts have almost exclusively focused on how agent-specific factors, such as dialogue structure and appearance, contribute to desired social outcomes (e.g., see Chattopadhyay et al., 2020). This overlooks the role of the physical environment shared by interactants in shaping such outcomes (Skjaeveland and Garling, 1997), which becomes salient in MR-based scenarios. To address this gap, the current study examines how evaluations of VHs are influenced by their interactions with the physical environment during social engagements.

As virtual surrogates of humans, a VH’s effectiveness and social potential is contingent on factors foundational to human’s face-to-face (FtF) interactions (Kopp and Bergmann, 2017). Like humans, VHs must be able to detect, discern, and respond to a multitude of cues to understand and convey context-appropriate behaviors during an interaction (Niewiadomski et al., 2010). Cues broadly constitute any sensory information (e.g., colors, setting, and dialogue) accessible in a communication environment (see Xu and Liao, 2020 for a review). Research to date primarily focuses on VHs’ detection and response to cues originating from the user, including the tone of voice (Moridis and Economides, 2012), facial expression, and posture (Vinayagamoorthy et al., 2006; Karg et al., 2013). At present, there is limited knowledge on how and when VHs can and should attend to cues originating from the user’s environment, which include cues within the immediate social context (e.g., pointing toward an object) as well as those outside (e.g., a phone ringing). These cues can provide important contextual meaning to a user’s behavior and can help shape context-appropriate behavior from the VH’s perspective. In this study, we have examined what we call *contextual responsiveness*: a VH’s capacity to detect and respond to cues occurring in the shared space between the user and the VH.

Contextual responsiveness may be an important design feature of VHs’ social and affective behavior toward users in MR because, unlike in virtual reality (VR), a VH is displayed in a user’s existing physical environment. As such, from a user perspective, the VH seems co-located in their physical space akin to FtF interactions, potentially rousing expectations about how the VH should behave and communicate given its access to contextual information in the shared space. While previous work has shown that human expectations of VH behavior vary based on factors such as VH appearance and task goals (Burgoon et al., 2016), a question that has been largely unexplored to date is whether and how a VH in MR—given situational awareness *via* real-world sensors—should respond to events and objects in the shared space (i.e., the user’s physical space).

Previous studies have shown that a VH’s nonverbal behaviors toward users can yield profound cognitive and affective impacts (Beale and Creed, 2009). For example, interactions with embodied agents that exhibited nonverbal feedback such as gestures were shown to engender more favorable evaluations (Bergmann et al., 2012; Krämer et al., 2013) and increase a sense of realism (e.g., Garau et al., 2005). Conversely, other works have shown that the absence of responsiveness to users can lead to unfavorable evaluations of a VH (e.g., Skarbez et al., 2011). Collectively, this work implies an expectancy of human-like responses from the VH to the user. Given that social interactions seldom exist in a vacuum, this expectation presumably extends to the shared environment such that the presence of contextual responsiveness may affect people’s feelings and beliefs about the VH and the social interaction.

One social interaction outcome that may be affected by a VH’s contextual responsiveness is social presence, or the subjective sense of “being with” another person (Oh et al., 2018). Social presence is an important factor in technology-mediated communication as it 1) contributes to the perception of artificial entities as social beings and 2) is associated with favorable outcomes such as enjoyment and social influence (cf. Oh et al., 2018). While there is limited consensus on the conceptualization and measurement of social presence (Parker et al., 1978; Bailenson et al., 2001; Nowak, 2001; Biocca et al., 2003), for the purposes of this study, we delineate social presence across two dimensions: copresence and connectedness. Copresence is characterized by a sense of being in the same space as another human, virtual, or otherwise, as well as the perception of mutual awareness and attention from others (Zhao, 2003). Connectedness refers to affective and relational evaluations of the VH, such as interpersonal closeness and mutual understanding; it has conceptual overlap with affinity, intimacy, immediacy, and attentiveness (cf. Manstead et al., 2012, p. 149).

From a theoretical standpoint, a VH’s contextual responsiveness is likely to influence copresence as it signals awareness of being in the same space with the user; a VH’s response to an event in the user’s physical space may help suspend the disbelief that the space is not actually shared and the VH is not really there with them. Should a VH not respond to events or objects in the shared space, a user may fail to sustain the illusion that the VH is copresent with them. Indeed, emerging work suggests that a VH’s contextual responsiveness to cues in a user’s physical space affects their copresence in MR. For example, when interacting with a virtual character in MR, users rated the experience as less plausible and felt a lower sense of spatial presence, a known correlation to copresence, when the character ignored a visible event in the background (i.e., a person walking by; Kim et al., 2020). Similarly, other studies have explored VH contextual responsiveness to static physical objects (e.g., Kim et al., 2017), moving objects (e.g., oscillating fan; Kim et al., 2019), and physical events (e.g., a wobbly table; Lee et al., 2016), finding mixed results.

As it relates to connectedness, human emotional responses are significantly affected by how a VH responds to the user (Ravaja et al., 2018), whereas their responses to objects and spaces are less influential. For example, Andrist et al., (2012) found that likeability and trustworthiness of a VH did not significantly change based on its ability to shift attention to objects in the shared environment. Similarly, the capacity to influence real-world objects, such as hitting a physical ball with a virtual golf club, failed to significantly influence emotional responses to a VH (see Schmidt et al., 2019). These findings imply that during a social interaction, a VH’s natural contextual responses to physical objects in the shared space may not significantly influence users’ affective response, although they may contribute to cognitive evaluations of the interaction, namely, copresence.

In discussing the future of MR-based collaborations, Podkosova and Kaufmann (2018) argued that “a strong sense of copresence is desirable in all types of scenarios” (p. 2). Scholars acknowledge a myriad of factors that shape copresence, although the effects of an agent’s contextual responsiveness to physical events remain largely unknown, a problematic reality considering the increased use of VHs in dynamic real-world spaces (see Powell et al., 2020). To address this gap, the present study investigates the extent to which a VH’s contextual responsiveness affects people’s sense of copresence and connectedness with the VH in the context of a collaborative task. Users are paired with a VH that either contextually responds to or ignores an event occurring in the physical environment. The experiment, thus, disambiguates the effects of contextual responsiveness on affective (e.g., likeability) and cognitive evaluations (e.g., copresence) and expands upon existing work by ensuring sufficient statistical power and using a control condition to examine the added value of contextual responsiveness. Moreover, results will help clarify the design requirements of VHs, thereby assisting in more effective creation and integration of VHs across a variety of social contexts.

## Materials and Methods

### Experimental Design

The primary goal of this experiment was to examine the extent to which a VH’s responsiveness to events occurring in the user’s physical environment influences a sense of copresence and connectedness to the VH. A 2 × 2 mixed design was implemented with physical event occurrence (yes/no) as a within-subjects factor and VH’s response to a physical event occurrence (yes/no) as between-subjects factor. The study was approved by an external ethics committee (Western IRB, now Wcg IRB), and all participants provided written informed consent.

### Participants

A convenience sample from a large technology company in the United States was recruited *via* internal communications. Based on a power analysis conducted using G*Power (Faul et al., 2009), a minimum sample of 56 participants was deemed necessary for the detection of a small effect size. In total, 65 participants (41 male) completed the experiment (M_age_ = 35.05, SD = 11.32), which took 15 to 20 min to complete.

### Apparatus

The experimental stimuli (i.e., the custom-built MR application) were deployed for use with the Magic Leap One (ML1), an optical see-through, head-mounted display (OST-HMD). The ML1 combines spatial mapping and digital light-field technology to superimpose 3D computer-generated imagery over real-world objects.

### Procedures

Upon arrival, the participants were welcomed, signed an informed consent, and were provided details about their participation in the study. To conceal the true objective of the experiment and prevent demand effects (Klein et al., 2012; Nichols and Edlund, 2015), participants were informed that they would be evaluating an early prototype for a collaborative MR application. Participants were to complete two consecutive sessions where they and a female VH partner would engage in a collaborative cube stacking task (see “MR Collaborative Task Application” section).

After explanation of the task by the experimenter and completing a self-contained tutorial using the HMD, the participants were presented with a visualization of the cube stacking arrangement they were to recreate with each VH. Before the collaborative task started, participants were “introduced” to the VH; the VH smiled, waved, and established eye contact. Then, a “loading” graphic appeared for 8 s, positioned above the table between the user and the VH. This loading screen had the sole purpose of creating sufficient and believable “waiting time” for the experimental manipulation to occur during the second session.

**FIGURE 1:**
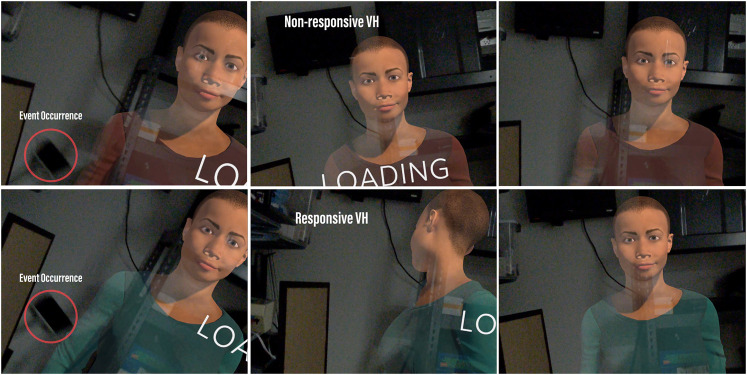
Visualization of the two experimental conditions wherein the VH either responded or ignored an event occurrence during the interaction.

**FIGURE 2:**
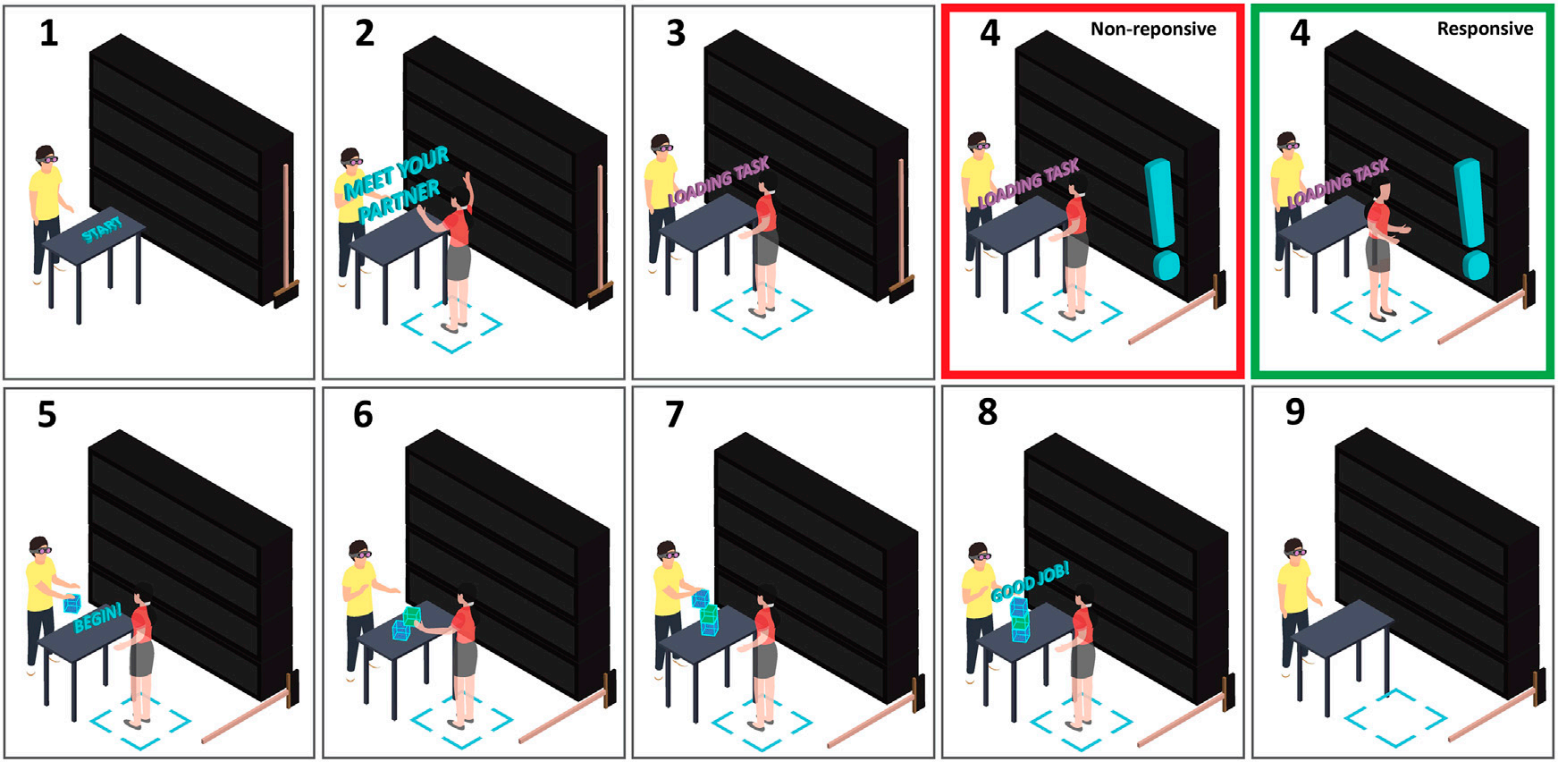
Graphic representation of the experimental procedures.

The first session was the same for all participants such that the entire interaction occurred without a physical event occurrence, functioning as a control condition. In the second session, the experimental manipulation of contextual responsiveness took place; a physical event occurrence (a broom falling) occurred behind the VH during the task loading screen. In the nonresponsive condition, the VH maintained mutual gaze with the participant, ignoring the event. In the responsive condition, the event triggered a nonverbal behavioral response (dubbed “contextual responsiveness”) by the VH, who turned her head in the direction of the fallen broom behind her (see Figure 1). After each trial, participants filled out a questionnaire, and after the second trial, suspicion on the true purpose of the experiment was gauged. Finally, participants were debriefed. A visualization of the entire experimental procedure, including the task and responsiveness manipulation, is shown in Figure 2.

## Materials

### MR Collaborative Task Application

A custom MR collaborative task application was created for use with the ML1 and was developed using Unity 3D software. During the task, the VH and participants faced each other with a table in between them. Participants relied entirely on hand gestures (e.g., hand wave and thumbs up) to interact with the VH and the other virtual content; they were presented with a short tutorial on how to pick up and place virtual cubes with their hand. All nonverbal user inputs triggered VH behavioral responses during the interaction: user head positioning determined attentional gaze, detection of a hand wave gesture triggered a reciprocal greeting from the VH, and successful placement of a cube on the table signaled the VH’s turn to place a cube. Each task trial required the participant and VH dyad to take turns placing a cube according to the presented visualization.

### The Virtual Human

**FIGURE 3:**
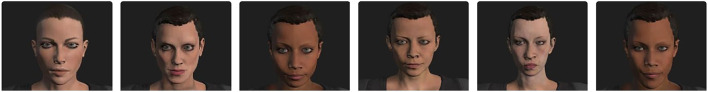
Six randomly generated VHs pretested prior to the stimuli development.

Appearance: To select a VH that is appealing to participants, we conducted a pilot study with six androgynous female characters. The VHs were created using Adobe Fuse; factors such as facial structure and skin color were randomly generated. Eight (4 females) participants rated still images of the VHs (Figure 3) in random order on dimensions of attractiveness and likeability, factors known to influence evaluations of virtual characters (e.g., Waddell and Ivory, 2015). The VH with the highest score on both measures was then selected for integration into the application. Last, to differentiate the VHs across both trials, each was given a different colored shirt.

Behavior: The two VHs were rigged and animated within Mixamo and imported into Unity. Custom scripts were then created to provide the VH with natural nonverbal behaviors as they pertain to gaze and facial expressions. As gaze is an integral predictor of copresence (cf. Oh et al., 2018), the VH was programmed to engage in dynamic gaze behavior (e.g., 1- to 3-s intervals of gaze fixations, alternating between the user and the environment; cf. Admoni et al., 2014, p. 394). Moreover, gaze fixation would shift toward the user’s hands during detected gestures (e.g., wave). In addition to gaze, subtle and appropriate facial expressions were programmed into the VH throughout the interaction and during specific events such as greeting the user and upon successful task completion (happy and neutral; cf. Krumhuber et al., 2009).

### The Event Occurrence

For this study to appropriately evaluate VH responsiveness to real-world events, the event itself needed to be 1) plausible given the interaction context, 2) detectable by the participant, and 3) capable of being triggered by the experimenter unsuspiciously. In other words, we employed a Wizard-of-Oz experimental approach such that VH’s response was controlled by the experimenter, and all of the subjects believed that they were interacting with a real autonomous agent. Prior to each session, a large broom was placed near the back of the room (behind the VH) and propped up against a small lever, which was connected by invisible wire to a heavy magnet switch on the opposite side of the room. As the experiment took place in a cluttered storage room, containing dozens of devices, wires, and cables on shelves and closets, the location ensured that the event occurrence was both plausible and detectable by the participant.

The experimenter – who was not in participants’ line of sight – would listen for the audio cue triggered by the loading screen during the second trial and then lift the magnet switch, triggering the release of the lever and causing the broom to tip over across the room and onto an adjacent metal cabinet. This event created a loud noise and was also visible to the participant as it occurred behind the VH. Immediately after lifting the switch, the experimenter would press the trigger button on the hand controller, which was not used by the participant at any point, to trigger the VH response (animation) to the event. As previously mentioned, the VH would either respond to the event by looking behind them (responsive condition) or would ignore the event completely (nonresponsive condition). All materials (e.g., lever and switch) were out of participants’ line of sight throughout the entire experiment.

### Measures

#### Demographics and Technology Use

General demographic information, such as age, gender, and job role, was collected. Additionally, participants were asked to rate how often they engaged in various technology-based activities during a regular week using a 7-point Likert scale (1—never to 7—all the time). Activities included remote collaboration, use of MR, and use of virtual assistants, among others (see Table 1).

**TABLE 1:** Mean scores and standard deviations for technology use across experimental conditions.

	Nonresponsive VH	Nonresponsive VH
Remote collaboration	3.5 (2.19)	2.82 (2.27)
In-person collaboration	4.77 (1.47)	5.18 (1.13)
MR use	2.94 (1.49)	2.27 (1.15)
AR use	1.75 (1.14)	1.52 (0.83)
VR use	1.5 (0.88)	1.42 (0.66)
Video chat use	4.5 (0.8)	4.42 (0.87)
Text app use	4.5 (0.88)	4.73 (0.91)
Virtual assistant use	3.22 (1.54)	2.94 (1.56)

#### Copresence

The primary variable of interest was copresence. As contemporary measures of copresence and related phenomena have varied widely in their measurement, conceptualization, and validity (Parker et al., 1978; Bailenson et al., 2001; Biocca et al., 2001), several steps were taken to 1) clarify its conceptualization, and 2) use a validated measure of the construct appropriate for an MR context. In this study, we conceptualized copresence as a multidimensional construct that comprises spatial copresence (sense of shared space) and mutual attention and responsiveness. Affective relational components previously used in other scales of copresence (e.g., connectedness and liking) were omitted to be able to clearly interpret and distinguish it from similar constructs. Based on this conceptualization, we developed a questionnaire which underwent three iterations, the largest consisting of an online study (N = 400) and confirmatory factor analysis (CFA). These investigations led to the copresence scale (see Table 2), a 15-item questionnaire consisting of 5-point Likert scale items (1—completely disagree to 5—completely agree) assessing participants’ level of agreement with various items, including “I felt that I was in the same space as the other person” and “The other person responded to my actions.” More information on the development of the scale is available upon request.

**Table 2:** Items comprising the Copresence questionnaire used in the experiment.


1. I felt that I was in the same space as the other person
2. It felt like the other person was with me
3. I felt that the other person and I were together in the same space
4. I felt that the other person and I were sharing the same physical space
5. I felt that I was in the presence of the other person
6. I felt that the other person paid attention to me
7. I felt that the other person responded to my nonverbal expressions (e.g., gestures, facial expressions)
8. I felt that the other person responded to shifts in my movements (e.g., posture, position)
9. The other person responded to my actions
10. I Felt that the other person was attentive to what I was doing
11. I Think that the other person noticed what I was paying attention to
12. The other person did not acknowledge my presence
13. The other person did not react to my behavior
14. I Felt that the other person was distracted
15. I Felt that the other person did not give their attention to me

Copresence was measured after the first (T1) and second (T2) interaction with the VH (Cronbach’s *α* = 0.91 and 0.93, respectively). Subsequent measures of connectedness, plausibility, and liking were only measured at T2 due to the investigation’s focus on evaluative differences in copresence between groups resulting from contextual responsiveness manipulation.

#### Connectedness

Connectedness was measured at T2 *via* a 16-item, 5-point Likert scale assessing participants’ sense of connection and mutual understanding with the VH (interpersonal closeness). Although this construct is often included in ever-expanding definitions of copresence (e.g., Biocca et al., 2001), we contend that connectedness is an affective, relational construct that is different from copresence. Participants indicated their agreement with 16 items on a 5-point scale (1—completely disagree to 5—completely agree), including “I could tell how the other person felt” and “I felt emotionally disconnected from the other person” (Cronbach’s *α* = 0.94).

#### Plausibility Illusion

Plausibility illusion (Psi) was measured at T2 to assess the extent to which participants felt that the interaction with the VH was actually happening (Slater, 2009). Previous work suggests that the perceived realism of a VH is influenced by whether its interaction with the physical environment is plausible (see Kim et al., 2017). Thus, this measure was included as it provides a barometer for the perceived credibility of the VH as being part of the physical environment. Psi was measured *via* a 9-item, 5-point Likert scale assessing participants’ level of agreement (1—completely disagree to 5—completely agree) with various statements, including “I had the feeling that the interaction was really happening even though I knew that some aspects of the environment were not real” and “I had a sense that the other person was part of the real environment even though I knew (s)he was not real” (Cronbach’s *α* = 0.9).

#### Liking

To assess the degree of positive evaluations of the VH (e.g., liking and trust), participants were asked to rate their level of agreement (1—complete disagree to 5—completely agree) with six statements about the VH at T2. These statements included “I like the other person” and “The interaction was pleasant” (Cronbach’s *α* = 78).

## Results

### Participants

In total, 65 participants took part in the study. Due to a technical error with the survey tool at the onset of data collection, items measuring liking and trust of the VH (5 items) were not displayed for the first 18 participants. Thus, only 47 participants’ responses to these items could be analyzed. The overall sample consisted of 41 males (63.1%), and the participants were between 19 and 64 years old (M = 35.05 and SD = 11.32). Participants did not significantly differ in their use of technology; *F* (1, 63) = 3.77 and *p* > 0.05.

### Manipulation Check

To confirm that users recognized and detected both 1) the disturbance and 2) the VH’s response, participants were asked their level of agreement (1–7) with a statement about the sense of the VH being aware of what was happening in the environment. This item was included in the questionnaire after each VH interaction across participants in both the responsive and nonresponsive conditions. A one-way analysis of variance (ANOVA) revealed no significant differences between conditions after the first interaction (control), M_responsive_ (*N* = 33) = 3.67 and *SD* = 0.89; M_nonresponsive_ (*N* = 32) = 3.41, *SD* = 1.01, *F* (1, 63) = 1.22, and *p* = 0.27. After the manipulation, however, there was a significant difference in perceived awareness of the VH between conditions such that participants in the responsive condition reported a stronger feeling that the VH was aware of the physical environment, M_responsive_ = 4.64 and *SD* = 0.70; M_nonresponsive_ = 3.44, *SD* = 1.22, *F* (1, 63) = 23.92, and *p* < 0.001. Thus, the manipulation of contextual awareness was successful.

### Suspicion Check

After completing the final questionnaire, participants were asked to verbally describe, in their own words, the purpose of the experiment. Participant responses varied, with some mentioning that the experience was designed to aid in enterprise team building or to optimize general collaborations in MR. While some participants in the responsive condition noted that the VH responded to the event, none explicitly articulated that this was the purpose of the study.

### Dependent Variables

#### Copresence

**FIGURE 4:**
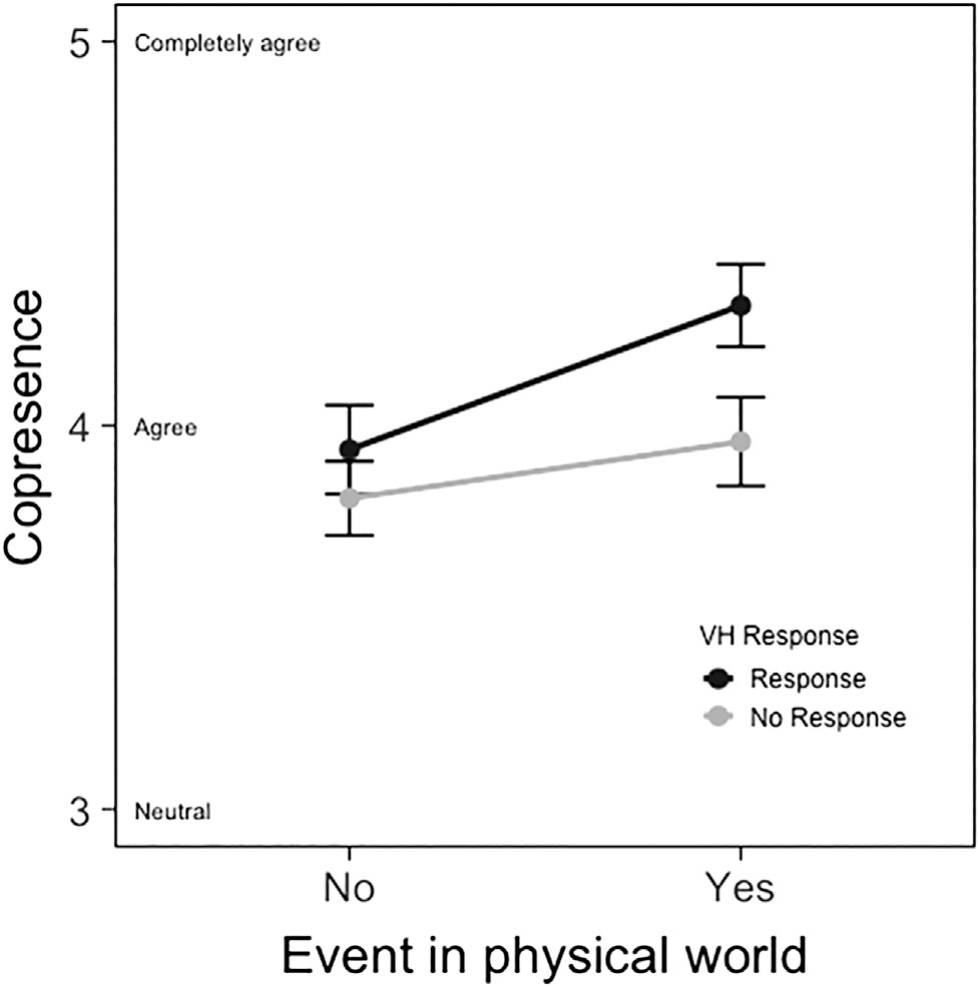
Graph demonstrating the interaction effect between time and contextual responsiveness on copresence with a VH.

**FIGURE 5:**
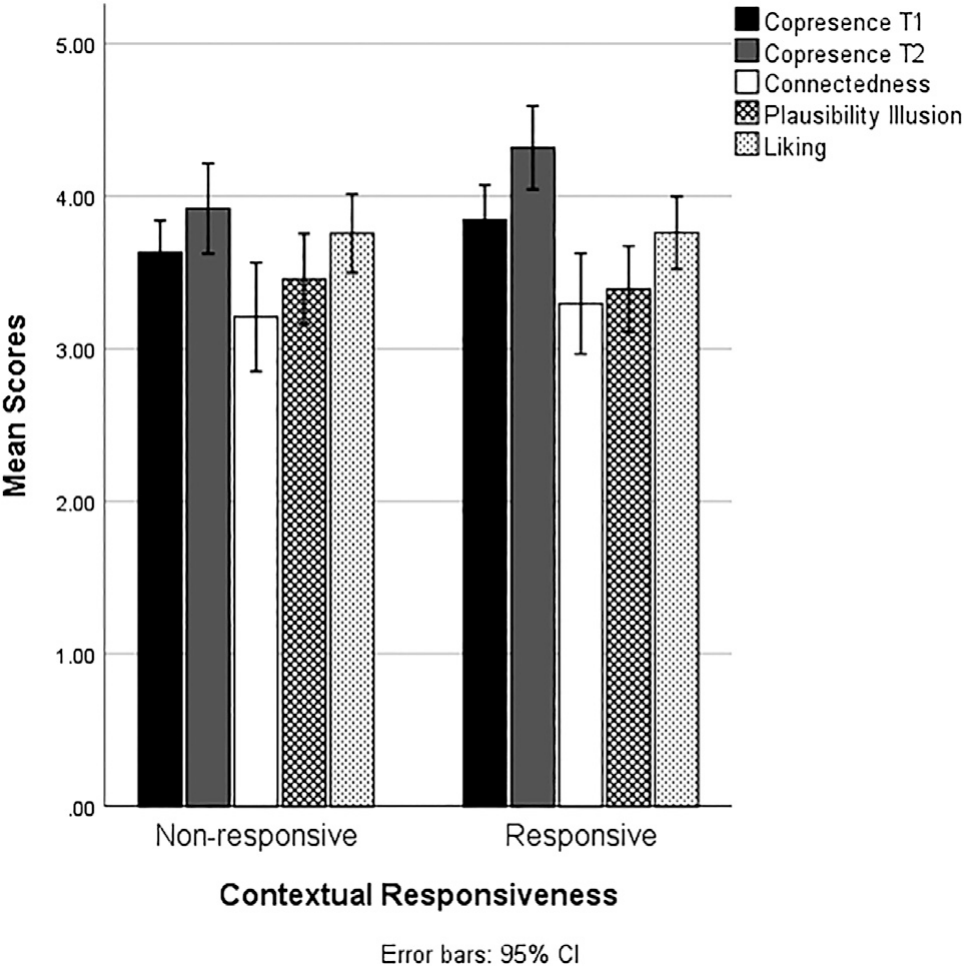
Bar graph demonstrating mean scores of various dependent measures across experimental conditions.

To test whether contextual responsiveness influenced copresence, a mixed ANOVA with event occurrence (yes vs. no) as the within-subjects factor and VH responsiveness to the event (responsive vs. non-response) as the between-subjects factor was conducted. Results demonstrated that there was a main effect of time, *F* (1, 63) = 40.25, *p* < 0.001, and partial η^2^ = 0.39, indicating that the average feeling of copresence increased after the second interaction with the VH. As expected, there was a significant interaction effect between event occurrence and contextual responsiveness, *F* (1, 63) = 7.62, *p* = 0.008, and partial η^2^ = 0.11 (see Figure 4). VHs who did not respond to the event occurrence during the second interaction (T2) elicited significantly less copresence (M_nonresponsive_ = 3.96 and *SE* = 0.12) than VHs who responded to the event (M_responsive_ = 4.31 and *SE* = 0.11; see Table 3); *F* (1, 63) = 5.06 and *p* = 0.02. See Figure 5 for a graphical representation of the effects of VH responsiveness on copresence and other dependent variables below.

**Table 3:** Mean scores and standard deviations across experimental conditions for key-dependent variables.

	Nonresponsive VH	Responsive VH
Copresence T1	3.67 (0.49)	3.8 (0.56)
Copresence T2	3.96 (0.65)	4.31 (0.62)
Connectedness	3.19 (0.79)	3.32 (0.69)
Plausibility illusion	3.12 (0.69)	3.45 (0.59)
Liking	3.76 (0.61)	3.76 (0.55)

#### Connectedness

A series of one-way ANOVAs were conducted on each dependent variable (Bonferroni-corrected alpha 0.05/3 = 0.017). The tests showed no differences between the responsive and nonresponsive condition in connectedness; M_responsive_ (*N* = 33) = 3.32 and *SE* = 0.12; M_nonresponsive_ (*N* = 32) = 3.19, *SE* = 0.14, *F* (1, 63) = 0.49, and *p* = 0.49.

#### Plausibility Illusion

With regards to Psi, contextual responsiveness did not significantly contribute to the perceived plausibility of the VH interaction; M_responsive_ (*N* = 33) = 3.45 and *SE* = 0.11; M_nonresponsive_ (*N* = 32) = 3.31, *SE* = 0.11, *F* (1, 63) = 0.722, and *p* = 0.39.

#### Liking

Last, with regard to liking, contextual responsiveness failed to significantly influence the likability of the VH; M_responsive_ (*N* = 23) = 3.76 and *SE* = 0.11; M_nonresponsive_ (*N* = 24) = 3.76, SE = 0.12, *F* (1, 45) = 0.001, and *p* = 0.98.

## Discussion

This study investigated the cognitive and affective implications of one particular building block of VH social intelligence: contextual responsiveness. Participants who interacted with a VH that nonverbally responded to an event in the shared environment with the user reported higher levels of copresence than those interacting with a VH who ignored the event. Note that this effect is robust, not only due to its effect size but also power, especially given that during the first collaboration session (which served as a control condition), copresence was already high due to features of the interaction (doing a task together, mutual gaze, and realism). The fact that we found an additional effect of VH responsiveness to the physical world lends credence to the power of this feature. With regards to affective evaluations, participants’ connectedness and liking of the VH did not differ based on the VH’s contextual responsiveness. Broadly, the results support the notion that evaluations of a VH in MR can vary as a function of their contextual awareness and response to objects in the user’s physical space—even when the object is not directly related or relevant to the task or interaction context.

Overall, the current work provides a modest contribution to VH research as it highlights the evaluative implications of this simple feature: users’ perception of a VH differs as a result of responsiveness, but only as it relates to cognitive evaluations (copresence), not affective evaluations (connectedness). We believe these results establish contextual responsiveness as an important factor, along with other visual and emotive factors (see Beale and Creed, 2009), capable of shaping social perception of VHs. Moreover, these results can help create more effective contextual design of VHs by establishing baseline relationships between VH responsiveness and copresence in a neutral, collaborative context. In the following sections, we discuss the theoretical and applied implications of the findings further and highlight avenues for future work.

### Cognitive and Affective Implications

#### Dimensionality of Social Presence

Conceptualization and measurement of social presence lacks consensus in HCI research, with studies varying in their treatment of the variable as a uni- or multidimensional construct. Indeed, contemporary theories of presence suggest social presence is a purely affective evaluation and that nonaffective (or cognitive) evaluations of interactants are subsumed under the spatial presence construct (Schubert, 2009). Our results demonstrate that when interactions occur in MR (with presumably uniform levels of spatial presence), cognitive evaluations of a VH, which are present in multidimensional scales of social presence, vary based on contextual responsiveness. Contrary to other current conceptualizations (Oh et al., 2018), our findings imply that copresence (a cognitive evaluation) can and should be disentangled from other constructs like connectedness (an affective evaluation) that have been increasingly included in the definition of social presence. This unidimensional distinction regarding copresence is particularly important considering a recent work in MR-based interactions with VHs which have leveraged social presence scales combining affective and cognitive items without parsing out the differences across those dimensions (e.g., Rzayev et al., 2019). In sum, we suggest that social presence itself has related yet independent cognitive and affective components, which we conceptualized as copresence (being in the same space and mutually aware) and connectedness (interpersonal closeness and mutual understanding).

Definitions of social and copresence have been used interchangeably and varied in scope and focus, ranging from a sense of the other person being “real” (Bailenson et al., 2001; Bailenson et al., 2004), to copresence as a relational construct (immediacy, intimacy) that is used for interactions with the outcome of feeling more connected to one another (Biocca et al., 2001; Harms and Biocca, 2004). Copresence and affective evaluations toward (virtual) humans have different determinants, as evidenced by the null effects of contextual responsiveness on connectedness. This provides a strong basis for our contention that copresence is a relatively neutral concept characterized by shared space and attention, which should be disentangled from outcomes that could be—but not necessarily are—a result of co-occurring phenomenon to copresence (for a similar argument, see Manstead et al., 2012).

#### Common Grounding

It is also important to address potential psychological mechanisms responsible for the direct effect of responsiveness on copresence. One explanation may be that contextual responsiveness creates common ground. When interactants perceive they have similar access to information or knowledge, this creates common ground (Clark, 1996). Common ground can be established through verbal and nonverbal behavior constructed from “whatever cues [users] have at the moment” (Olson and Olson, 2000, p. 158), with copresence being one of eight primary cues used by interactants to obtain common ground (Clark and Brennan, 1991). In the context of this study, seeing a VH detect and respond to an event grounded in one’s physical reality seemingly contributed to copresence by anchoring the VH in the physical space, establishing common ground.

### Should Virtual Humans Be Responsive?

Intuitively, contextual responsiveness seems like a desirable feature for VHs, especially if cultivating a sense of copresence is the ultimate goal. Indeed, emerging work around VHs is creating them with the capacity to sense the real world (e.g., Randhavane et al., 2019). However, responsiveness is not merely binary, rather it is a multifaceted concept that can vary in accuracy and magnitude, among several other dimensions. Each of these aspects of responsiveness can have differential effects on the user experience. For example, one study found that the magnitude of a VH’s behavioral response (blushing) significantly increased copresence (Pan et al., 2008). Other experiments provide evidence that responsiveness, even if it increases copresence, can have a negative effect on other important outcomes depending on the difficulty of the collaborative task. For example, when learning recipes from a virtual assistant, the addition of nonverbal communicative cues increased copresence at the expense of task performance (Kontogiorgos et al., 2019). Responsiveness may be a double-edged sword depending on how and when the user is able to process the behavior (see Admoni et al., 2014) and whether responsiveness occurs prior to or during a particular task. As the behavioral response in our study occurred prior to the task, and the task itself was relatively simple, we did not assess task completion time. However, future work should investigate the effects of VH responsiveness across task-types, difficulty, and settings.

Our results also highlight how contextual responsiveness may be paired with Internet of Things (IoT) sensors and actuators to discern complex signals from the environment and trigger VH actions that maximize copresence and benefit user experience. IoT sensors are increasingly enabling accurate detection and classification of physical events (e.g., door slams, footsteps, and voice) occurring in social spaces (see Wang et al., 2019). This level of environmental awareness affords VHs the capacity to respond to disturbances with realistic accuracy in MR, a potential requirement considering previous work showing errors in VH behaviors negatively affect perceived interaction quality (Skarbez et al., 2011). Furthermore, recent work overlaying VHs onto robotic actuators have enabled dynamic interactions with users, such as moving physical board game pieces during play (Lee et al., 2019). While this “physicality” feature contributed toward copresence with the VH, it is unclear whether gains in copresence are comparable to those gained through less elaborate setups, such as those employed in this study. As cost increases with the complexity of VH physicality, future work should engage in cost-benefit analyses of such features, thereby clarifying the requirements of VHs in specific contexts, such as how precise contextual responses should be to maximize copresence.

### Ecological Validity and Generalizability

From a methodological perspective, our study sought to address a prevailing concern associated with VH research in a controlled lab environment: the sterility inhibits naturalistic (random) events to occur, which are part of real social environments. As Sandini et al., (2018) note, “realistic testing of architectures for social intelligence…in unstructured natural living spaces enable the understanding of advantages/weaknesses and foster innovation towards development of socially cognitive [agents]” (p. 15). In instantiating a detectable and plausibly random event occurrence, we were able to examine human responses to VH behavior as would be expected in real-world scenarios. This ultimately bolsters the ecological validity of our study, although generalizability is largely relegated to dyadic collaborations involving a task-irrelevant event or disturbance (e.g., phone call and knock on the door).

### Limitations

There are several caveats to acknowledge when interpreting the results from this study. For one, our experiment tested the effects of a VH’s response to a single benign event occurrence in an enterprise context (office building). Generalizing our findings to complex social environments saturated with many such occurrences (e.g., shopping malls) should be done cautiously. Additionally, it is important to note that potential limitations associated with the study’s use of a mixed within-between design. While the order of the trials was not counterbalanced and order effects cannot be entirely ruled out, the use of randomization and inclusion of a control condition mitigate such issues. Moreover, as the primary focus of this work was to examine whether responsive VHs elicit greater copresence than nonresponsive VHs, it is unlikely that the results are significantly affected by the order of the trials given that both between-subjects conditions were exposed to the same within-subjects condition first (control). Another limitation relates to the duration of the interaction. Exposure to the VH during each collaborative task lasted roughly 1 min. While short encounters with VHs may be common in certain contexts (e.g., information kiosks), the effects of responsiveness on copresence during longer interactions remain unclear. We emphasize the importance of future work to examine the implications of a VH’s perceptual bandwidth: are multiple instances of contextual responsiveness in a busy environment distracting? Are there ceiling effects of contextual responsiveness on copresence?

## Conclusion

The current work explores how VH behavior, namely, the capacity to detect and respond to physical events occurring in the user’s environment, influences interpersonal affect and cognitive evaluations of a VH in MR. In doing so, we extend research on the determinants of copresence beyond user- and technology-centric factors, such as mutual gaze and attractiveness, respectively. Our findings also contribute to theories of presence; contrary to recent conceptualizations (e.g., Oh et al., 2018), our results suggest that copresence can and should be disentangled from other constructs that have been included in the definition of copresence as a multidimensional concept. Nonetheless, copresence remains a desired outcome for social interactions with robots (e.g., Herath et al., 2018) and VHs alike (e.g., Strojny et al., 2020), and this investigation highlights how contextual responsiveness aids in facilitating copresence. In testing the effects of contextual responsiveness in a collaborative MR setting, we also establish avenues for further research into situational (collaborative vs. competitive task) and contextual factors (familiar vs. unfamiliar space) that may shape users’ affective and cognitive evaluations of VHs.

The spectrum of human activities will only continue to involve VHs as realities blend and MR devices grow in popularity. If indeed users “expect a VH to behave like a real human” (Lee et al., 2019, p. 7), our findings suggest that this expectation is met at least in part through contextual responsiveness. It is evident that this factor merits further attention from industry and academic research teams alike, and we hope this investigation helps establish clearer requirements for VHs and social robots in collaborative real-world settings.

## Data Availability

The raw data supporting the conclusions of this article will be made available by the authors, without undue reservation.
